# Mental health recovery, goal setting and working alliance in an Australian community-managed organisation

**DOI:** 10.1177/2055102918774674

**Published:** 2018-05-14

**Authors:** Grenville Rose, Lorraine Smith

**Affiliations:** 1 University of New South Wales Sydney, Australia; 2 Flourish Australia, Australia; 3 The University of Sydney, Australia

**Keywords:** goal setting, mental illness, recovery, self-efficacy

## Abstract

This article examines the relationships between goal setting and achievement, working alliance and recovery in an Australian mental health community-managed organisation. The study gathered data over a 14-month period after the introduction of routine outcome measures. Both goal achievement and the strength of the working alliance were shown to have a positive effect on the personal recovery of the clients in the study. Both working alliance and goal achievement are robustly supportive at whatever point a person is on in the recovery journey. The brief goals card used is a useful adjunct to other tools.

## Introduction

The non-government community mental health support services (known in Australia as community-managed organisations (CMOs)) sector is a large and growing provider of mental health services in Australia. Between 1992 and 2011, expenditure on CMO services as a percentage of state and territory spending, which is the bulk of health spending in Australia, grew from 2.1 to 9.3 per cent of funding (Department of Health and Ageing, 2013). If the Australian government adopts recommendation 9 of the National Review of Mental Health Services (National Mental Health Commission, 2014), then the sector will expand further in the future. Further impetus to expansion of the CMO sector is being provided by the introduction of the National Disability Insurance Scheme (NDIS) into the mental health sector. The NDIS is a market-based system for service delivery (Disability Reform Council, 2015) and market-based CMOs, as well as private service providers, are expected to have a larger role in service provision after the transition to the NDIS is complete (Bateman and Henderson, 2016).

CMOs seek to assist people to live sustainably better lives in the community: empowered, in control and meaningful; however, without rigorous evaluation of outcomes achieved, it is impossible to say whether the organisations are achieving good outcomes for the people accessing their services. Similarly, knowing the elements of the support that promote meaningful outcomes is crucial to service improvement.

CMOs are becoming more evidence based in their approach and more CMOs are incorporating and evaluating routine outcome measures into their services. However, the people working in CMOs have generally lower levels of training than other mental health professionals such as psychologists, psychiatrists or mental health nurses and, surprisingly, less than half of CMOs in Australia have formal training in the outcome measures used in their services (Australian Mental Health Outcomes and Classification Network (AMHOCN), 2013). The problem posed by this lack of training is exacerbated by the administrative complexity of outcome measures that are commonly used. Research conducted by the team that developed the Collaborative Recovery Model, for example, has previously found that the volume and complexity of the forms used for that tool is one of the barriers to successful use (Marshall et al., 2010).

Exploratory qualitative and quantitative work conducted across a broad range of clients and staff in a national Australian CMO also suggested that there is a significant proportion of staff and clients who would benefit from having a goal setting tool that is linguistically simpler and contained on a single form, or card (Rose et al., 2015). This is particularly so as a significant percentage of people supported by CMOs in Australia have severe and chronic mental illness which is sometimes associated with cognitive impairment, and thus there may be scope for the development of less language intensive tools for use in these services. Australia is also a nation with a great deal of cultural diversity, approximately 28 per cent of people living in Australia were born in an Anglophone country (Australian Bureau of Statistics, 2016b) and may therefore benefit from working with simpler forms that require a lower level of English literacy to comprehend.

A robust factor in promoting mental health recovery and well-being is effective goal setting. There is strong evidence that working towards clearly defined goals that the person has set for themselves improves outcomes across a wide variety of illness states and therapy types and helps to build and strengthen the therapeutic alliance (Clarke et al., 2009; Schrank et al., 2012; Smith et al., 2011). Goal setting works best when the person who is working towards the goal chooses the goals they want to achieve. There is also substantial evidence that self-efficacy plays a role in goal achievement. The more confidence an individual has in their capacity to undertake and execute a task, the more likely they are to be successful. Low levels of confidence are associated with low goal achievement (Mann et al., 2013; Smith et al., 2011).

Psychological research on common factor theory suggests that fidelity measures and standardisation of delivery of a particular therapeutic technique may be less important than how the person sees their working alliance with the ‘therapist’ and how engaged the therapist is with whatever technique or system they, and the people they support, feel is useful (Messer and Wampold, 2002; Wampold, 2015). Even under tightly controlled conditions, recovery outcomes vary considerably based on the healthcare professional working with the person (Anderson et al., 2009). In a community setting, with far less tightly controlled conditions than a clinical trial, and with any intervention being conducted by support workers with lower levels of training than most mental health professionals, it may be more useful to focus more on robust factors that work under a variety of conditions. In this setting, the working alliance or trust relationship has been shown to be important to people receiving support (Huxley et al., 2009), and a reliable component of people’s recovery (Horvath and Greenberg, 1989).

Mental health CMOs in Australia are recovery focused rather than symptom reduction focused. While there is no universally agreed upon definition of mental health recovery, a construct that has been used in the development of a recovery scale in Australia (Hancock et al., 2015) contains the elements that will be used as the working definition of ‘mental health recovery’ used in this article. The elements are as follows:

- Engaging in valued activity;
- Having a future focus;
- Not being dominated by symptoms and feeling connected to friends and community.

**Figure 1.**
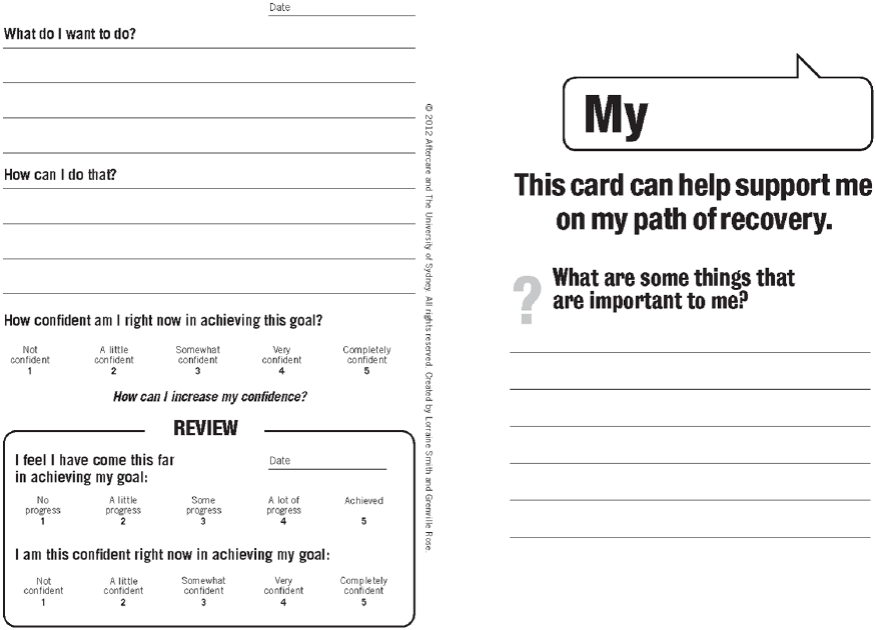
Example of a goal card (front and back pages – other side is the same as the back page).

A useful adjunct to current tools, therefore, would be a simple goal setting process that is a user-friendly, flexible tool that can be tailored to the individual’s needs and capabilities. To investigate the applicability of a simple goal setting process in community mental health settings, a goal setting card was developed by the authors based on work done at the University of Sydney that has been previously trialled in a number of physical health states, as well as being trialled for administration by both clinicians and non-clinicians (Smith et al., 2011, 2014; Figure 1).

Importantly, this card was designed to assist people to set goals that related to what they wanted to do, rather than the goals health professionals wanted them to set.

In May 2014, a national mental health CMO implemented a set of client outcome measures as a part of routine outcome measurement (ROM) to aid in the development of better services. In addition, a goal setting tool, a simple goals card, was rolled out as part of the service delivery. The University of Sydney, Western Australian Association for Mental Health (WAAMH) and the CMO undertook a study to track the utility of goal setting and the therapeutic alliance using the ROMs collected by the service organisation.

The aims of the study were to answer the following questions:

1. To what extent did goal setting contribute to improvements in recovery and working alliance?
2. What types of goals were set, how many, and how many were achieved?
3. What was the relationship between clients’ confidence scores and goal achievement?
4. What was the relationship between working alliance and recovery?

## Method

Ethics approval was gained from the Human Research Ethics Committee of the University of Sydney (Ref: #14599).

Sample: Clients registered with the CMO for mental health support and had signed consent forms to have their de-identified data used for research purposes on commencing support with the organisation were eligible to take part in the study. A total of 57 sites provided data and these were spread among regional and metropolitan New South Wales and Queensland Australia. The quantitative goal setting, recovery and working alliance instruments were completed at two time points, the first and last completed of each measure, as a part of regular support in community outreach programmes for people with mental health issues. Initial forms completed were not therefore necessarily completed at service initiation as most people were already receiving services from the organisation.

### Instruments

The data were collected from the support worker and the clients using paper-based forms. The scores and descriptive text were then entered into the Carelink Plus database (Icon Global, 2015). The data were gathered between May 2014 and June 2015. All support staff received brief internal training in the administration of the outcome measures and in goal setting through the organisation’s learning and development department. The instruments used were as follows:

1. An A6 card format goals card developed by the authors in consultation with staff and clients of the service (Rose et al., 2015). Linguistically simple and contained on a single card rather than on multiple forms. Scales used were 5-point scales reported as means, with the exception of goal achievement which was collapsed to either goal achieved or goal not achieved.
2. Working Alliance Inventory short form (WAI), both client and ‘therapist’ versions were used (Hatcher and Gillaspy, 2006; Horvath and Greenberg, 1989) scored on a 7-point scale from never to always. A higher score indicates stronger alliance after reverse coding negatively worded questions. Scores were converted to a score out of 100 for ease of interpretation. This measure was chosen due to the robust relationship of the working alliance with mental health outcomes (e.g. Wampold, 2015) and the nature of the work performed in CMOs in which the focus is more on the working relationship rather than specific interventions.
3. Recovery Assessment Scale–Domains and Stages (RAS-DS) (Hancock et al., 2015). This was scored on a 4-point scale where a higher score indicates a higher level of recovery and converted to a 100-point scale according to the formula given in the manual (Hancock et al., 2014). This scale was used as it measures a number of domains of recovery, is an accurate measure of a wide range of recovery outcomes, and has found meaningful acceptance by both staff and consumers as an aid to collaborative goal setting (Hancock et al., 2015).

### Analysis

The results were analysed using SPSS 23 (IBM Corporation, 2014). Scores on each of the above measures were calculated for the person’s first and last completed measure. To assess the effect on the outcome of achieving a goal, analysis of variance (ANOVA) was conducted on the WAI and RAS-DS. Regression to the mean occurred in both the WAI and RAS-DS across time and as the scores on those measures are unreliable covariates by definition, rendering analysis of covariance (ANCOVA) unsuitable, the initial scores were divided into blocking factors and entered as factors in the ANOVAs with the final score as the dependent (Tabachnick and Fidell, 2001). Independent *t* tests were conducted to test for baseline differences between those who had achieved a goal and those who had not. Descriptive statistics were calculated on the types of goals, how many were set and how many achieved.

To test for the effect of the working alliance on recovery, partial correlations were calculated between the final RAS-DS score and final WAI controlling for initial client and support worker scores and initial RAS-DS score.

Overall recovery change scores were calculated for the RAS-DS and WAI and one-sample *t* tests were conducted on client and support worker scores, adjusted for the regression to the mean effect using the correlation between times 1 and 2 to calculate the percentage of change in the direction of the mean that was due to regression to the mean (Trochim, 2006), and then adjusted for familywise error using the Holm–Bonferroni stepdown procedure. Repeated-measures ANOVA was used to assess differences in the perceptions of the alliance between support workers and clients over time. Possible confounds were tested for by calculating correlations of relationships between length of time in the service, time between completion of first and last measures and working alliance or recovery scores.

## Results

Data were gathered across metropolitan and regional NSW and Queensland. As the information was collected in a naturalistic setting within an operating Australian CMO, the *N* is different for each of the instruments collected. This was largely due to the preferences of the people accessing the services for using one instrument over another. If the focus of support and recovery is developing rapport and establishing an effective working alliance, then it is problematic to make the use of specific forms compulsory. There is thus a wide variation in the *N* for each instrument collected.

A total of 704 people had completed the RAS-DS for the first and last measure. Demographics are shown in Table 1.

**Table 1.** Demographics of participants.

Age	Range, 18–79; mean 45^a^
Gender	425 F (60.4%); 275 M (39.1%); 1 not stated (%); 3 missing
Country of birth	Australia 77%; non-English-speaking countries (19.5%); unknown 3.5%^b^
Aboriginal or Torres Strait Islander	6%^c^
Primary diagnosis	Schizophrenia (24%); depression (25%); bipolar disorder (10%); anxiety disorder (11%); other (30%)

a Older than the Australian average age of 38 years.

b Representative of the Australian population (Australian Bureau of Statistics, 2016b).

c Higher percentage than in the Australian population (Australian Bureau of Statistics, 2016a).

The median length of time if the clients had been in the service at the conclusion of data collection was 861 days, with the range being from 28 to 10,407 days. Preliminary relational analyses showed that length of time receiving support from the service, or length of time between completing measures was not significantly related to changes in working alliance or recovery scores over time (*p* > 0.05), and thus this analysis is not presented. Overall average scores for both working alliance and recovery were very high, 91 and 72 out of 100, respectively, but each showed only 1 per cent change, on average, over the 6 months of the study (Table 2).

**Table 2.** Initial and final scores out of 100 on questionnaires.

Questionnaire	*N*	Mean	SD
WAI-Client Rated Initial	620	84.4	13.0
WAI-Client Rated Final	486	84.7	12.5
WAI-Support Worker Rated Initial	639	80.8	12.0
WAI-Support Worker Rated Final	506	81.2	12.4
RAS-DS Total Score Initial	704	72.1	14.0
RAS-DS Total Score Final	704	73.2	13.9

WAI: Working Alliance Inventory; RAS-DS: Recovery Assessment Scale–Domains and Stages; SD: standard deviation.

### Goal achievement, recovery and working alliance

**Figure 2.**
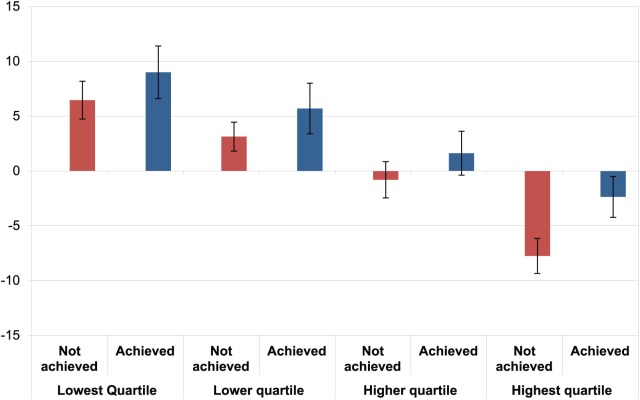
Change in recovery score by goal achievement and baseline RAS-DS quartile, means and standard errors.

Initial screening of the data indicated that those who had a high initial score on the RAS-DS had lower scores at follow-up, whereas those who had low initial scores had improved their scores on follow-up. To account for this regression to the mean effect, the scores were divided into quartiles based on the baseline RAS-DS score. Results of the ANOVA showed that those who had achieved a goal had a significantly higher final level of recovery, *F*_1,293_ = 7.259, *p* < 0.01, $\eta_{p}^{2}=0.025$. The partial eta squared indicates a small to medium effect (Miles and Shevlin, 2001). Furthermore, the effect of goal achievement was not significantly affected by regression to the mean as there was no interaction effect between the goal achievement and the initial score blocking factor, *F*_3,291_ = 0.094, *p* > 0.5, $\eta_{p}^{2}=0.001$ (Figure 2).

In regard to the interaction of goal setting and working alliance, people who went on to achieve goals had a stronger working alliance score at baseline than those who did not, both for the support worker and client rated scales, *t*_269_ = 2.69, *t*_272_ = 3.89, respectively, *p* < 0.01.

### Goals set and achieved

There were validly recorded and reviewed goals for 295 people. Of these, 70.5 per cent (188) were still working towards a goal and had reviewed their goals at least once and 34.4 per cent (104) had fully achieved at least one goal. For three people, the data were missing. The number of additional people who are working towards a goal but who have not yet reviewed their goals is unknown as only data for reviewed goals cards were included in the database. A goal audit conducted at the half-way point in the study suggests that the achievement rate was not impeded by the specificity of the goals with an average of 83 per cent of goals being rated as moderately or highly specific by two independent raters, whose ratings were strongly correlated *r* = 0.92. In that analysis, 28 per cent of goals related to relationships and socialising, 23 per cent related directly to mental health symptomatology and strategies, 14 per cent to housing and 17 per cent to health. Other goals referred to finance, independent living skills and employment (Rose et al., 2015).

### Goal achievement and confidence

There were 165 people who had completed the confidence scales at each time point. Baseline confidence was higher in people who went on to achieve a goal compared to those who did not, 2.9 out of 5 compared to 2.2, respectively, *t*_164_ = 4.2, *p* < 0.01. ANOVA showed that having already achieved a goal did not increase confidence in achieving goals in the future compared to those who had not achieved a goal, *F*_1,104_ = 2.0, *p* > 0.05, $\eta_{p}^{2}=0.02$. However, those who had higher confidence at baseline also had higher confidence at final measure, *F*_1,104_ = 11.25, *p* < 0.01, $\eta_{p}^{2}=0.01$. Examination of the interaction between confidence and goal achievement found that the people who had low initial confidence had higher final confidence if they had achieved a goal compared to those who had low confidence and did not achieve a goal. Intriguingly, those who had high confidence initially had lower final confidence if they had achieved a goal compared with those who did not, *F*_1,104_ = 4.71, *p* < 0.05, $\eta_{p}^{2}=0.06$. Table 3 shows the change in scores by each initial score quartile.

**Table 3.** Confidence and goal achievement (maximum of 5).

Baseline confidence level	Goal achievement	Mean	Standard deviation
Lower 50% at baseline final score	Did not achieve goal	1.46	0.69
Lower 50% at baseline final score	Achieved a goal	2.14	1.15
Upper 50% at baseline final score	Did not achieve goal	2.48	0.82
Upper 50% at baseline final score	Achieved a goal	2.3	0.88

### Working alliance and recovery

There were 420 matched pairs of clients and support workers completing both the WAI and RAS-DS at two time points. Repeated-measures ANOVA showed that support workers tended to score the alliance slightly lower than clients, 83.48, 81.28 out of 100, respectively, *F*_1,985_ = 22.8, *p* < 0.05, $\eta_{p}^{2}=0.025$, and that difference was consistent across time points. The correlation between the support workers’ scores of the alliance and clients’ scores are reasonably strong when first completed, *r* = 0.61 (*p* < 0.01), but weakened to *r* = 0.43 (*p* < 0.01) at the second time point.

To investigate the effect of working alliance on recovery, partial correlations controlling for initial WAI scores of both client and support worker as well as initial RAS-DS score were calculated between the final RAS-DS score and the final WAI client and support worker scores. The result shows small to medium effects of *r* = 0.316 and *r* = 0.233, respectively.

As previously stated, the RAS-DS and WAI scores were affected by regression to the mean. The initial extreme scores tended towards the mean over time. At the extremes, there was statistically significant change that was not attributable to the regression to the mean effect. The change scores for each quartile are shown in Table 4.

**Table 4.** Change scores for each quartile.

Initial score quartile	RAS-DS (38.5%)^a^	WAI Support Worker (55.2%)^a^	WAI Client (40.3%)^a^
Lowest	9.04*	8.57*	10.33*
Second lowest	2.59	1.23	0.35
Second highest	−0.23	−1.92	−0.77
Highest	−5.94*	−6.09*	−5.56*

WAI: Working Alliance Inventory; RAS-DS: Recovery Assessment Scale–Domains and Stages.

a Percentage of change attributable to regression to the mean (Trochim, 2006).

* Statistically significant one-sample *t* test (p < 0.05), adjusted for regression to mean effect and adjusted for familywise error within each quartile group using Holm–Bonferroni stepdown.

## Discussion

Overall, goal setting and achievement contributed to improved outcomes for people and was significantly related to a strong working alliance. The number of goals set and achieved was in line with that reported in other health contexts and were more often set around socialising and relationships than specifically to address mental health symptomatology. The relationship between confidence and goal achievement was complex. Those who were initially confident were more likely to achieve a goal, but having achieved a goal did not necessarily translate to higher confidence in further goal achievement.

It is well established that when a person achieves a goal it is associated with better recovery (Clarke et al., 2006, 2009) and this was well demonstrated in this study. Importantly, however, the increase due to goal achievement in this study was not strongly related to the stage of recovery of the person when they first started setting goals using this method. Achieving a goal was associated with a higher level of recovery compared with having not achieved a goal. This is in contrast to the overall recovery scores which showed a strong effect of regression to the mean over time for the people in the service. Focusing on helping the service users to achieve a goal, this study strongly suggests, is a reliable way to ensure that any person being supported by the service will have the best opportunity to have better results wherever they are on their recovery journey. The strength of goal achievement is in line with previous research in physical health (Gardner et al., 2016; Mitchell et al., 2011; Smith et al., 2007).

In regard to goal setting and working alliance, those who did go on to achieve a goal had a stronger working alliance at the start of the study period. That those with a stronger initial alliance were more likely to achieve a goal suggests that there was a stronger collaboration on goal setting, and this suggests that working alliance and goal achievement should be a part of routine outcome reviews along with mental health recovery for the provision of the most appropriate support environment or support person. The effect of working alliance on recovery was similar to those obtained in earlier studies (Howgego et al., 2003; Martin et al., 2000). The high client scores on the WAI raise the issue of whether the client and worker are each giving higher scores than they might in a completely anonymous setting. While the scales were rated independently the small differences between the client and worker scores on average, and the relatively low correlations suggest that, while this is a limitation that must be borne in mind while using this type of tool, that it need not be fatal to the process and that establishing reasonably honest and direct feedback on the working relationship could prove to be a valuable tool in itself.

There was, however, a strong regression to the mean effect in relation to the recovery measure. Those who had a higher level of recovery at the start of the study period had lower scores on average, whereas those with lower initial scores improved. Not all of the change in either case was attributable to regression to the mean, but this raises the question of whether people may be screened at regular intervals using an instrument such as the RAS-DS and may then be streamed to offer support that is the most appropriate to their present situation. It is unfortunately beyond the scope of this study to suggest best kind of support, or indeed to ascertain whether appropriate stepped support exists, for people those who scored highly on the recovery measure who therefore feel unwell but are perhaps not unwell enough to be in need of the highly personalised support given in Australian CMOs. Any cut-off points and procedures for directing people to the most appropriate support based upon their initial RAS-DS scores could be further explored beyond the blunt division into quartiles which were used here for the purposes of analysis. However, this study points the way towards a more efficient service that is focused on supporting the specific people who will benefit from their services.

The low number of people achieving goals may be a reflection of the length of time it can take to achieve some goals, and that these are affected by factors such as goal specificity, degree of difficulty, level of confidence and degree of training in implementing goal setting. Although there was a generally strong working alliance and the great majority of goals set were moderately or highly specific, the tool was introduced along with a suite of other tools and the training time allotted to goal setting was less than 2 hours in a group setting. Future implementations, or continued use in the current setting, would benefit from more goals related training. This would assist with greater engagement with goal achievement and therefore increase goal achievement.

People who had higher initial confidence in achieving their goals were more likely to go on to achieve a goal. This finding accords with the general literature on goal setting (Mann et al., 2013; Smith et al., 2011). However, the average levels of confidence in achieving goals were low, those who did not achieve goals had an average confidence of only 2.2 out of 5 and those who achieved a goal only 2.9. Furthermore, those who had low initial confidence but still achieved their goal received a boost in confidence, whereas the confidence of those who had higher initial confidence and achieved a goal showed lower confidence in achieving future goals. This suggests not only regression to the mean as a component, but also that initial confidence need not be very high. Much of the literature suggests, however, that high confidence is essential to effective goal setting; it is perhaps the case, in the setting of this CMO at least, that confidence should be allowed to be as low the support worker and the service user see as befitting the specific aims and goals of the person. This must be done, of course, while bearing in mind the other factors that contribute to successful goal setting, such as specificity, achievability and appropriate strategies (Bandura and Locke, 2003; Smith et al., 2007). Goals, additionally, should be altered and adjusted as an active process between the support worker and the person accessing their services. The robustness of goal setting in improving well-being makes the case for the extra resources devoted to goal setting training. All people using the services can potentially benefit.

## Limitations

A limitation to this study was that it was done in a naturalistic setting as a result of a service introducing a new suite of measures and procedures. This means that the baseline measure was not a person’s initial measure on entry to the service, but the measure that was first completed by them after the introduction of the suite. In addition, although the instruments were to be administered in 3-month intervals, due to the nature of the services, the length of time between administrations varied. Nevertheless, the analysis showed that length of time receiving services and length of time between data collection had no significant impact on the findings regarding recovery, working alliance and goal achievement. Yet gathering baseline data on entry to the service would have been a more robust measure of the effect of the service.

## Conclusion

Collaborative goal setting is a robust method of support for people with mental health issues, and a simplified goal setting tool can be successfully used in the Australian mental health CMO setting even with minimal training. Similarly, a strong working alliance has also been demonstrated to be a robust component of mental health recovery. This study has demonstrated that in the varied and less tightly controlled environment of a mental health CMO that each of these methods can be successfully employed to support people with mental health issues and suggests that regularly screening people for stage of recovery and working alliance may lead to more efficient and effective service delivery. Measuring the working alliance between service user and support worker may also enhance the effectiveness of the service.
